# Moving Breast Cancer Therapy up a Notch

**DOI:** 10.3389/fonc.2018.00518

**Published:** 2018-11-20

**Authors:** Erik W. J. Mollen, Jonathan Ient, Vivianne C. G. Tjan-Heijnen, Liesbeth J. Boersma, Lucio Miele, Marjolein L. Smidt, Marc A. G. G. Vooijs

**Affiliations:** ^1^Department of Radiotherapy, GROW School for Oncology and Developmental Biology, Maastricht University, Maastricht, Netherlands; ^2^Department of Radiation Oncology (MAASTRO), Maastricht University Medical Centre+, Maastricht, Netherlands; ^3^Division of Medical Oncology, Department of Surgery, Maastricht University Medical Centre+, Maastricht, Netherlands; ^4^Division of Medical Oncology, Department of Internal Medicine, Maastricht University Medical Centre+, Maastricht, Netherlands; ^5^Department of Genetics, Louisiana State University Health Sciences Center, New Orleans, LA, United States; ^6^Stanley S. Scott Cancer Center, Louisiana State University Health Sciences Center, New Orleans, LA, United States

**Keywords:** breast cancer, Notch, personalized precision treatment, treatment resistance, resensitisation

## Abstract

Breast cancer is the second most common malignancy, worldwide. Treatment decisions are based on tumor stage, histological subtype, and receptor expression and include combinations of surgery, radiotherapy, and systemic treatment. These, together with earlier diagnosis, have resulted in increased survival. However, initial treatment efficacy cannot be guaranteed upfront, and these treatments may come with (long-term) serious adverse effects, negatively affecting a patient's quality of life. Gene expression-based tests can accurately estimate the risk of recurrence in early stage breast cancers. Disease recurrence correlates with treatment resistance, creating a major need to resensitize tumors to treatment. Notch signaling is frequently deregulated in cancer and is involved in treatment resistance. Preclinical research has already identified many combinatory therapeutic options where Notch involvement enhances the effectiveness of radiotherapy, chemotherapy or targeted therapies for breast cancer. However, the benefit of targeting Notch has remained clinically inconclusive. In this review, we summarize the current knowledge on targeting the Notch pathway to enhance current treatments for breast cancer and to combat treatment resistance. Furthermore, we propose mechanisms to further exploit Notch-based therapeutics in the treatment of breast cancer.

## Introduction

### Breast cancer

Breast cancer is the second most common malignancy, worldwide (1). Breast cancer screening and early detection has increased, leading to better outcome. Furthermore, a number of treatment options, have improved survival (2). First line therapies include surgery, radiotherapy, and systemic treatment (including: chemotherapy, endocrine therapy and targeted therapy). Treatment options for breast cancer exist in the neo-adjuvant (prior to surgery) and adjuvant setting (after surgery).

There is strong evidence that tumors that respond to neo-adjuvant chemotherapy with a pathological complete remission (pCR) have improved long-term prognosis (3). Conversely, tumors that do not respond to neo-adjuvant chemotherapy have a higher chance of recurrence. Adjuvant therapy targets remaining (micro-metastatic) cancerous cells, thereby preventing recurrent disease.

Clinical choices for the type of systemic treatment are guided by expression of estrogen receptor (ER), progesterone receptor (PR), and Human Epidermal Growth Factor Receptor 2 (HER2) in tumor biopsies, in concurrence with TNM classification, tumor grade, and age. However, it is widely accepted that breast cancer is a heterogeneous disease, from primary tumor to metastatic sites (4). Gene expression profiling (involving hierarchical clustering) has had a significant impact on classification of breast malignancies. Molecular breast cancer subtypes revealed different clinical behaviors and retain distinct differences in biological mechanisms (Table 1)—associated with tumor aggressiveness, metastasis, and response (8, 10–18, 22). Based on gene expression profiling, the MINDACT trial has shown that tumors with genomic “low risk” features do not require chemotherapy. This included some node-positive tumors irrespective of molecular subtype (23). Similarly, the TAILORx study has recently shown that early stage, node-negative breast cancers with low or intermediate recurrence scores do not benefit from adjuvant chemotherapy and can be treated with endocrine therapy alone (24). Additionally, gene expression analysis showed a sub-classification in Triple Negative Breast Cancer (TNBC) into at least 4 molecular subgroups (12, 19, 25) with observed differences in response to chemotherapy (20), by providing more detailed information inter-tumor heterogeneity (26). The combination of histological and genetic classification of each tumor will further guide therapy selection and disease outcome (26, 27) and, ultimately, form the basis for personalized precision medicine.

**Table 1:** Subtype classification of breast cancer.

	**Normal breast-like**	**ERBB2^+^**	**Basal-like**	**Luminal subtype A**	**Luminal subtype B**	**Luminal subtype C**
Specific gene signature	•Low to absent gene expression of the ER •Adipose tissue and other nonepithelial cell type gene expression •Strong expression of basal epithelial genes and low expression of luminal epithelial genes	•Low to absent ER gene expression •High ERBB2 pathway genes expression including; ERBB2 and GRB7 •Intermediate expression of luminal-related genes and proteins (e.g., ESR1 and PR) and low expression of basal-related genes and proteins (e.g. keratin 5 and FOXC1) •Enriched with high frequency of APOBEC3B-associated mutations •1 out of 5 patients of HER2^+^/HR+ tumours will be identified as non-luminal	•Low to absent gene expression of the ER-related genes, intermediate expression of HER2- related genes •High expression of proliferation, suppression of apoptosis, cell migration and/or invasion genes •High expression of (HMW) keratins 5, 6, 14, and 17, laminin, and FABP7 •BRCA1 mutations associated, none of the BRCA1 tumors showed evidence of ERBB2 amplification •Unique entity in breast cancer, more similarities with other cancer types •“stem/progenitor” cell phenotype •Multiple basal (TNBC) subtypes and mixed clinical response;. •TNBC often includes Claudin-low subtype.	•Highest expression (of luminal subgroups) of the ERα gene, X-box binding protein 1, trefoil factor 3, hepatocyte nuclear factor 3 a, and estrogen-regulated LIV-1 •vs. Luminal B: lower grade, lower number of mutations across the genome, lower number of chromosomal copy-number changes (e.g., lower rates of CCND1 amplification), less TP53, higher PIK3CA and MAP3K1, higher/similar GATA binding protein 3 •Subgroup shows HER2 amplification/overexpression	•Low to moderate expression of the luminal specific ER-related genes •vs. Luminal A: higher expression of proliferation/cell cycle-related genes or proteins—lower expression of several luminal related genes or proteins (e.g. lower progesterone) •Subgroup of Luminal B is found hypermethylated. •Subgroup shows HER2 amplification/overexpression	•Low to moderate expression of the luminal specific genes including the ER cluster •Separated from luminal A/B due to a subset of genes with unknown coordinated function, shared with Basal-Like and ERBB2^+^ tumors
Notch activation (mRNA), (protein)		•High Notch 2 expression	•High Notch 2 expression	•High Notch 2 expression	•High Notch 2 expression	•High Notch 2 expression
		•Notch 4 expression, positively correlates with ER positivity.	•Triple negative tumors express high Notch1 ,2 & 3	•Notch 4 expression, positively correlates with ER positivity.	•Notch 4 expression, positively correlates with ER positivity.	•Notch 4 expression, positively correlates with ER positivity.
		•Notch 1 expression is inversely correlated with HER2 expression	•Absence of ER expression correlates with higher Notch 3	•Notch 1 expression is inversely correlated with HER2 expression	•Notch 1 expression is inversely correlated with HER2 expression	
		•Absence of ER expression correlates with higher Notch 3	•Notch 1 expression in 100% of TNBC cases assessed (6)	•High Notch 3 expression compared to TNBC (7)	•Notch 1 expression inversely correlated with ER & PR expression (5)	
		•Notch 1 expression inversely correlated with ER & PR expression (5)	•Notch 4 expression in 73% of TNBC cases assessed (6) •Low Notch 3 expression in TNBC compared to Luminal A (7). •Notch 1 enriched in basal sub-type (5).	•Notch 1 expression inversely correlated with ER & PR expression (5)		
TP53	33%	70%	80%	10–15%	30–40%	80%
PIK3CA	–	39%	9%	45%	29%	–
Similarities		Highest mutational loads (Basal-Like > ERBB2^+^ > Luminal)		•Expression of the estrogen receptor separates luminal from non-luminal •Luminal expression signature: ER1, GATA3, FOXA1, XBP1, and cMYB. •Proliferation/cell cycle-related (e.g., RB1 and Cyclin D1) and luminal/hormone-regulated pathways •More diverse mutational profile		
			•Basoluminal subtype; distinguishable subtype based on heterogeneous CK5/14 expression, Laakso et al. (8) and Haughian et al. (9).			
Clinical outcome		•Worst/poor prognosis •Shorter disease free survival, earlier development of distant metastases •Shortest survival •Suggested not to benefit much from endocrine therapy •HR status shows predictive value for pCR •HR– increased 5yrs relapse	•Worst/poor prognosis •Shorter survival •Similar to Luminal B at 10 years. •Suggested not to benefit much from endocrine therapy •Poly-chemotherapy is only effective treatment— variable between subtypes	•Best prognosis •Lowest rate of local or regional relapse. •vs. Luminal B: less chemo-sensitive (multi agent)	•Intermediate prognosis	•Worst prognosis luminal subtypes

*Representation of gene-expression analysis and clinical outcome of the multiple intrinsic subtypes of breast cancer. TP53/PIK3CA; percentage mutated cases. Data from: Sorlie et al. (10), Sorlie et al. (11); Laakso et al. (8), Cheang et al. (12), Prat et al. (13), Voduc et al. (14), The Cancer Genome Atlas (15), Prat et al. (16), Rakha et al. (17), Perou et al. (18), Lehman et al. (19), and Masuda et al. (20). Notch activation mRNA data: Touplikioti et al. (21). Protein Data: Speiser et al. (6), Dou et al. (7), Yuan et al. (5). HMW, High Molecular Weight; ER, estrogen receptor; HR, hormone receptor; PR, progesterone receptor; TN, triple negative; and TNBC, triple negative breast cancer*.

### Intra-tumor heterogeneity and tumor stem cells

Regardless of clinical or molecular subtype, intra-tumor heterogeneity is a common feature of all human solid tumors (28), and is a major determinant of treatment outcome in breast cancer (15, 29). Tumor growth is thought to be driven by small populations of cancer cells with self-renewal and multi-potential properties (30), coined cancer stem cells (CSC) (31). These CSCs are involved in malignant behavior (invasion and metastasis) and resistance to treatment (32). Thus, CSCs are of high clinical importance, and targeting CSC self-renewal appears necessary for obtaining a durable response. Furthermore, intra-tumor heterogeneity can be driven by mutation or deregulation of stem cell signaling pathways such as Notch, Wnt, Shh, and others as well as through the tumor microenvironment; including nutrient-, oxygen levels, and paracrine interactions with other cell types (fibroblasts, blood vessels, and immune cells) (33). Herein, Notch has shown interesting targeting opportunities in cancer (34).

### Notch

#### Notch signaling

**Figure 1:**
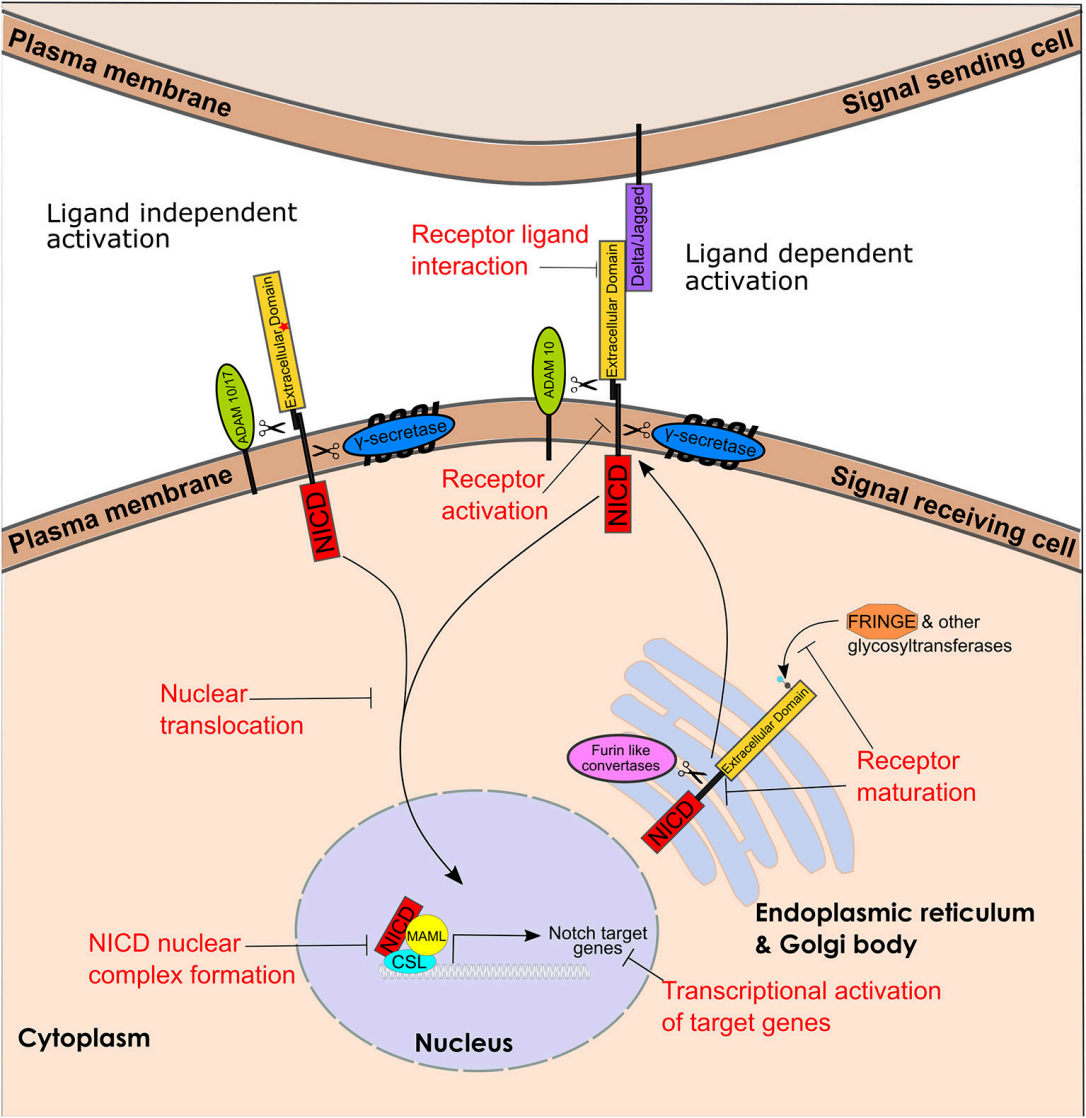
Notch receptor maturation, ligand dependent and independent activation pathway and targetable steps. Representation of Notch receptor maturation, ligand dependent and independent activation and the key enzymes involved. Text in red represents steps that can be targeted. The red star in Notch extracellular domain represents Notch activating mutations leading to ligand independent signaling. (CBF1, Su(H), Lag1), CSL; NICD, Notch intracellular domain and MAML, Mastermind-Like.

**Figure 2:**
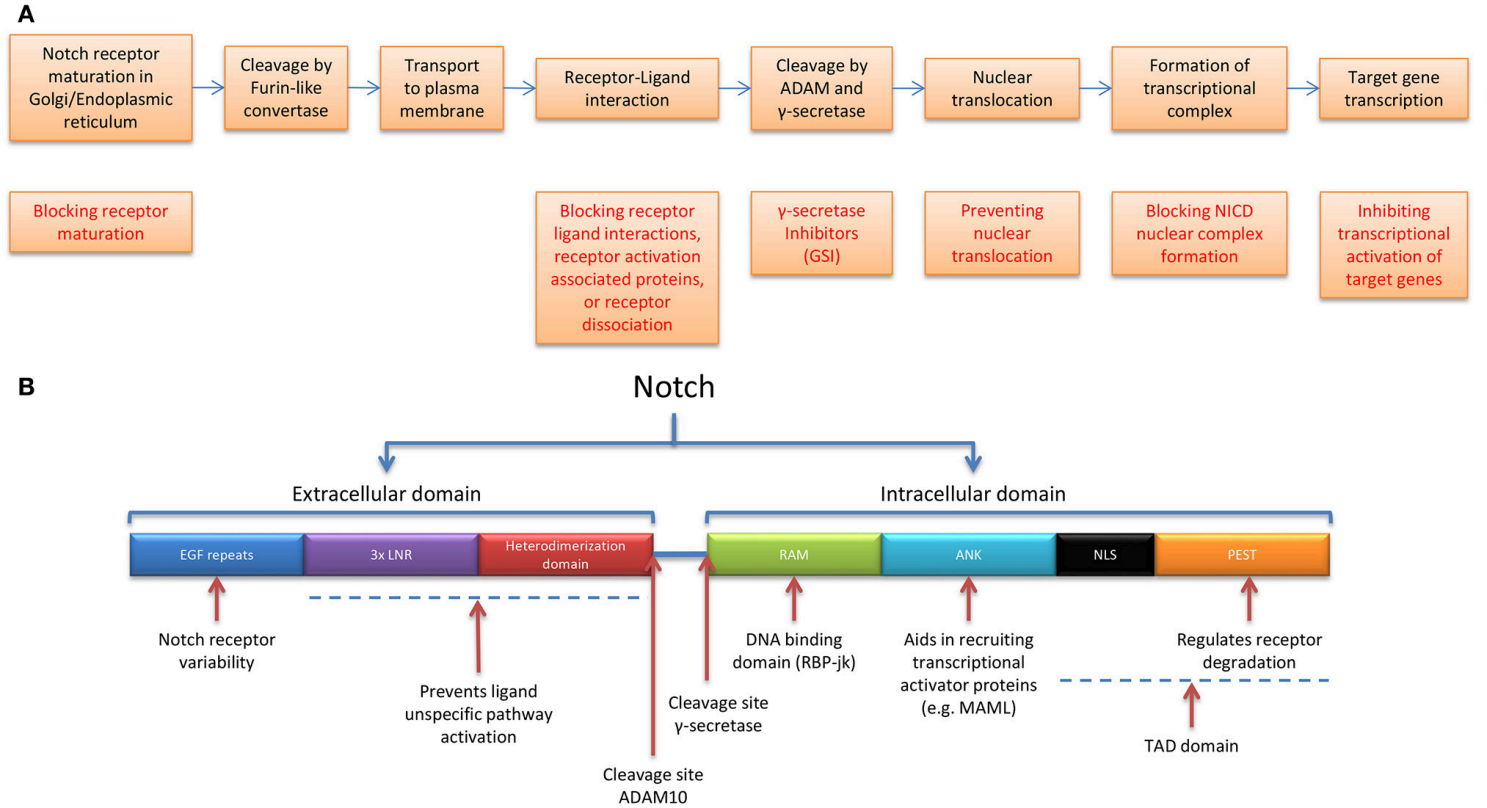
Notch receptor maturation and pathway activation, targetable options, and receptor functionality. **(A)** Stepwise representation of the process of Notch receptor maturation until receptor activation, followed by transcriptional output (not shown), and possibilities in targeting the Notch receptor pathway. **(B)** Notch receptor functional domains and corresponding functions ANK, Ankyrin repeats; LNR, Lin12-Notch Repeats and RAM, RBP-jk association module.

Notch signaling (Figure 1) is a cell-to-cell communication system of type I single-pass transmembrane Notch receptors (Notch 1-4) and transmembrane ligands (Delta/Jagged (JAG)). Notch receptor maturation starts in the Golgi/Endoplasmic reticulum. Glycosylation of Notch proteins in the Golgi and ER is known to play a role in the regulation of Notch activity (35). Fringe proteins can both positively and negatively regulate Notch ligands however the full scope of their roles in breast cancer are unclear (36). Furin-like convertases cleave the non-covalently associated Notch heterodimer, which is transported to the plasma membrane (Figure 2A).

The extracellular domain consists of epidermal growth factor (EGF)-like repeats, followed by a negative regulatory region (NRR) which includes 3 LNRs (Lin12-Notch repeats) and a heterodimerization domain which prevents receptor activation in the absence of ligand(37) The intracellular portion of Notch (NICD) also contains multiple regions and domains, the RBP-jk association module (RAM), Ankyrin repeats (ANK domain), and the TAD domain—which consists of Nuclear Localization Sequences (NLS) and the PEST domain (regulates receptor degradation) (38) (Figure 2B). Notch 1-4 have relatively short lifespans and undergo degradation through the ubiquitin-proteasome and lysosomal pathways. The PEST domain contained in the NICD is likely to play a role in E3 ubiquitin mediated turnover. In fact, mutations in the PEST domain of Notch 1-3 in TNBCs, have been shown to increase Notch half-life and lead to increases in Notch downstream targets. Promisingly TNBCs with these mutations have been shown to be sensitive to GSIs (39). Similarly, alterations in the tumor suppressor and ubiquitin ligase Fbwx7/cdc4 target the PEST domain of Notch (40).

The E3 ligase MDM2 has been shown to contribute to the degradation of Numb and through ubiquitination leading to activation of Notch in breast cancer. Treatment of MCF7 cells with drugs targeting the acidic domain of MDM2 showed a reduction in Notch signaling (41). Furthermore, ubiquitination of Notch1 by MDM2 has been shown to activate Notch rather than leading to degradation (42). MDM2 has also been shown to regulate p53 degradation through ubiquitination, which, along with its role in Notch regulation makes it an attractive target for drug discovery along with other E1-3 ligases and interacting proteins. Knockdown of the E2-conjugating enzyme UBC9 and inhibition of the E1 activating complex SAE1/UBA2 has also been shown to impair the growth of Notch1-activated breast epithelial cells (43). Pevonedistat (MLN4924), is an inhibitor of NEDD8, a ubiquitin like protein that can neddylate E3 ligases. Pevonedistat has been shown to induce apoptosis in MCF-7 & SKBR-3 cells in combination with 2-deoxyglucose (44), and to sensitize breast cancer cells to radiation *in vivo* (45). Bortezomib, an FDA approved proteasome inhibitor has been shown to inhibit multiple genes associated with poor prognosis in ERα breast cancer (46), however several clinical studies have shown contradictory results in advanced/metastatic breast cancer (47–49).

A number of components of post-translational modifications pathway have been implicated in regulating Notch stability including Fbw7, Itch, β-arrestin, Fe65 and Numb (50–53). Numb negative breast cancers have increased Notch signaling which can be reverted to basal levels with overexpression of Numb and visa-versa knockdown of Numb in Numb positive breast cancers leads to upregulation of Notch signaling (54). Further research into the mechanisms of Notch post-translational modifications and degradation may provide novel therapeutic targets as well as for other malignant diseases.

Signal transduction occurs through the Notch ligand on the “signal-sending cell” interacting with the Notch receptor on the “signal-receiving cell.” This interaction process involves two sequential proteolytic cleavage events – first by the ADAM10 metalloprotease which sheds the extracellular domain, leading to the release of the NICD. The γ-secretase complex is composed of 4 polytopic transmembrane proteins including a catalytic subunit the aspartyl protease presenilin (55) The sequential proteolysis activation mechanism is essentially the same for Notch1-3 receptors (56). The activation mechanism for Notch4 -although likely similar to the other Notch family members- has not been reported yet. The NICD then translocates to the nucleus where it forms a protein complex with CSL (Cbf-1/RBP-jk in mammals, Su(H) in Drosophila and Lag-1 in C. elegans) and MAML (Mastermind-like) and induces transcription of multiple Notch downstream target genes (38) (Figure 2B). Additionally, a number of non-canonical pathways have been described downstream of Notch, including transcriptional activation of ERα-dependent genes (57), and NF-kB (58), activation of the PI3K-AKT-mTOR pathway (59), and activation of mitochondrial metabolism (60, 61). Mammalian cells express four Notch receptors and five ligands in a highly tissue specific and content dependent manner (38). Activation levels of specific pathways within the global notch signaling pathway has been found to differ within mammary epithelial cells and this can lead to different phenotypic responses (62).

Notch can also be phosphorylated which can have contradictory effects depending on the number of cleavage steps it has undergone and the specific kinase involved in phosphorylation (63). Phosphorylation of Notch by glycogen synthase kinase 3 (GSK-3) can reduce Notch transcriptional activity & protein levels (64, 65) and may be a target for possible therapies. Site specific methylation of NICD1 has been shown to make it less stable than a methylation defective mutant (66) indicating other possible post-translational targets (67).

#### Targeting notch in cancer

In many solid tumors the Notch signaling pathway is deregulated or mutated (68), affecting most hallmarks of cancer (69). Notch gene expression is frequently deregulated in breast cancer (70) and shows extensive crosstalk with several major signaling pathways. Further, there is ample evidence for the diverse role of Notch signaling in tumor formation, progression, and resistance to treatment in breast cancer (71).

Due to the multi-step activation process, several Notch pathway interventions are being explored at the level of: I. blocking receptor maturation, II. receptor–ligand interactions, III. receptor activation associated proteins, IV. nuclear translocation, V. NICD nuclear complex formation, and VI. transcriptional activation of target genes (72) (Figure 2A). This includes, but is not limited to antibodies, small molecule inhibitors, and inhibitors of γ-secretase (GSI) (72, 73).

Despite the increased evidence for deregulated Notch in numerous malignancies and resensitization opportunities (34, 74–81), many clinical studies investigating Notch targeting are on hold or have been terminated. Notably, most of these trials were conducted in recurrent, heavily pre-treated chemo resistant cancers and used dose-limiting non-selective pan-Notch/GSIs. Additionally, because of a lack of biomarkers predicting outcome to Notch therapies, potential responders were thus not effectively selected [reviewed in (71)]. As a result, this has not led to effective interventions using Notch inhibitors combined with standard of care. Therefore, in this review, we have focused on the possible role of Notch in enhancing the efficacy of breast cancer treatment.

#### Notch and breast cancer

##### Role of notch in breast development.

The normal mammary gland experiences a period of rapid growth and development at puberty. Thereafter, and until menopause, it undergoes cycles of expansion and regression with each estrous cycle, pregnancy, lactation, and involution (82). This homeostasis requires stem cells and their existence was first demonstrated using transplantation experiments to reconstitute a functional mammary gland in rodents (83). Dontu et al. demonstrated the presence of early progenitor/stem cells capable of differentiating along all three mammary epithelial lineages (myoepithelial, ductal-, and alveolar epithelial). Gene expression analysis revealed similarities with progenitor and stem cell associated pathways, thereby identifying mammary stem cells (MaSCs) in 3D culture systems (84). More recently, *in vivo* imaging has identified bi-potent basal stem cells in the mammary gland, yielding both myoepithelial and luminal cells (85) and Notch plays a role in this process (86–89). Bouras et al., have performed extensive research on the role of Notch in MaSCs. In MaSCs, Notch1 is differentially expressed between subtypes (90), and its expression is higher in the luminal type cells (90, 91). Furthermore, Notch1/3 mark the luminal progenitor cells in mammary gland development (89, 91). Downregulation of Cbf-1/RBP-jk resulted in increased proliferation of MaSCs, thereby influencing absolute stem cell numbers. However, this proliferation resulted in increased and disorganized side branching, with increased number of end buds and basal cells in these end buds. Therefore, RBP-jk downregulation regulates the formation of a more basal cell phenotype. Additionally, overexpression of the endocytic protein NUMB, a negative regulator of Notch, produced the same effects. This shows that reduced Notch signaling is important in proliferation of the basal cell population and MaSCs. Conversely, increased levels of Notch1 in the luminal cells showed that constitutive Notch activation is important for commitment to the luminal cell lineage (High Keratin8/18, Stat5, and p63 downregulation) (90). Moreover, it has been reported that Notch4 is involved in promoting stem cell renewal of mammary epithelial cells (mammospheres) *in vitro* (92, 93), and is involved in stem cell activity (94)–possibly through JAG1 signaling (95) and PKCa-Notch4 interaction (96). Furthermore, Notch and p63 signaling guide the establishment of basal and luminal epithelial cells (97) and PTEN/JAG1 play an important role in mammary epithelial stem cells (98).

##### Role of notch in breast cancer development and metastasis.

The role of different Notch pathway components in breast cancer development has been extensively researched. Stylianou et al. showed that in many breast cancer cell lines Notch ligands, receptors, and target genes are aberrantly expressed (99). Charafe-Jauffret et al. identified a 413-gene CSC profile (including Notch2) using normal and malignant mammary tissue (100), identifying breast cancer stem cells (BCSCs) through ALDH^+^ (101). ALDH^+^ cells were capable of self-renewal, differentiation, tumor formation in mice, and showed increased metastatic potential. ALDH^−^ cells hardly generated tumors. Results from a Meta–analysis involving 3867 patients showed that Notch1 expression positively correlates with breast cancer progression and that higher expression is associated with a transition from ductal carcinoma *in situ* to invasive cancer. Furthermore notch1 overexpression was correlated with significantly worse overall and recurrence-free survival. The data further suggested that Notch inhibitors may be useful in blocking early progression of ductal carcinoma *in situ* (5).

Aberrant activation of the Notch signaling pathway has been shown to promote an aggressive phenotype partially through NF-κB, whereas de-activation of Notch signaling abrogates this aggressive phenotype (58). Furthermore, in TNBC, tumor cells activated NF-κB upregulates Jagged-1, which stimulates Notch signaling in CSCs (102).Tumor derived Jagged1 has been shown to be an important mediator of bone metastasis in breast cancer. Jagged1 activates stromal Notch signaling which in turn induces IL-6 secretion from osteoblasts stimulating tumor growth. Notch signaling also directly stimulates maturation of osteoclasts exacerbating bone metastasis. Destruction of bone matrices releases TGF-β upregulating Jagged1 in the tumor giving a positive feedback loop. GSIs treatment in turn reduces bone metastasis by targeting stromal Notch signaling (103).

*In vivo* studies using TNBC and ERα^+^ cell lines showed an association between Notch3 expression and distant metastases which was diminished in Notch3 null cells. This finding was corroborated using TNBC cells from a patient-derived brain metastasis (104). An *in vivo* study using a more metastatic variant of the HER2+ MDA-MB435 isolated from *in vivo* brain metastasis showed activation of the Notch signaling pathway. Inhibition of Notch using the γ-secretase inhibitor DAPT or knockdown using RNAi against Notch and Jagged2 resulted in inhibition of the migratory and invasive phenotype (105). Furthermore fewer brain micrometastases were found when Notch1 was silenced in an MDA-MB-231 model (106). Breast tumor cells in the brain highly express IL-1β which leads to surrounding astrocytes expressing Jagged1 which stimulates Notch signaling in CSCs (107). Oskarsson et al. showed that breast cancer cells that metastasize to the lungs enhance their ability to survive through expression of the extracellular matrix protein tenascin C. Tenascin C is associated with aggressiveness and pulmonary metastasis and enhances stem signaling components including Notch (108).

*Notch1*. Notch1 is aberrantly expressed in breast cancer (99) and high Notch1/4 mRNA expression and activity are associated with worse prognosis (30). In Ductal Carcinoma *in situ* (DCIS), Notch1 signaling is active and associated with the development of breast cancer (109). Both Notch1 and Notch4 are identified as common sites of proviral integration in mammary mouse tumors (110, 111), and induce mammary (MMTV)-tumors when overexpressed in transgenic mice (112–114). Larger studies have shown that expression of Notch1/4 and JAG1 is associated with poor prognosis in breast cancer (115). Moreover, JAG1 expression is an independent predictor of poor outcome in node-negative disease (116) and higher NICD1 expression correlated with sentinel lymph-node positive patients (117). Notch1 levels were progressively associated with the transition from DCIS to invasive basal cancer (5).

In human breast cancer, a meta-analysis including approximately 4000 cases showed that elevated Notch signaling is associated with increased disease recurrence (118). Pathway and network analysis revealed that altered Notch1 signaling occurred in ER^+^/PR^+^/HER2^+/−^ breast cancers (119), whereby Notch1 mutations are more prevalent in HER2^−^ than HER2^+^ tumors (120). JAG1-Notch signaling leads to Cyclin-D1 induction (121), a gene that is essential for normal breast development in mice (122) and frequently deregulated or amplified in human breast cancer (123, 124). k Notch1 activating mutations/rearrangements have also been observed in TNBC (in EGF repeats and NRR) (125) and in the basal-like phenotype (116). Additionally, Notch1 promotes stem cell maintenance through c-Jun signaling (126). Further, Reedijk et al. revealed that JAG1 is an independent predictor of poor outcome in multivariate-analysis (115) with other well-known outcome predictors (nodal metastases, patient age, tumor size, node status, ER positivity, and tumor grade) (5, 115, 127). Higher NICD1 expression correlated with sentinel lymph-node positive patients—strengthening Notch1's role in the metastatic process (117).

Leong et al. provided data that JAG1 and Notch1 are involved in epithelial-mesenchymal transition (EMT) through SLUG and E-cadherin. They showed that SLUG facilitated E-cadherin repression (through Notch1 inhibition) and inhibition of HEYL blocked tumor growth and metastasis, showing JAG1-Notch1-SLUG dependency (128). Furthermore, NICD1 expression negatively correlated with E-cadherin and showed increased invasive capacity of Notch1, (129). This was also the case under hypoxia with differences observed in high/low Notch signaling cell lines (130). Mechanistically, hypoxia-induced EMT is mediated through SLUG and SNAIL (131). A JAG2-EMT relationship has been shown too (through Notch1), revealing a broader spectrum of Notch1 activation and involvement in hypoxia and metastatic potential of CSCs (132). Additionally, high Notch1 and HIF predict a worse prognosis (133). These results show that Notch1 signaling is important for EMT and downregulation of E-cadherin, ultimately creating a more invasive phenotype. Furthermore, as described above, the invasiveness of the tumor and hypoxia induced EMT requires Notch1 signaling, demonstrating a hypoxia/Notch1/EMT axis. Thus, inhibition of Notch1 can be tumor suppressive by removing the inhibition on E-cadherin expression, regardless of hypoxia.

Downregulation of JAG1 or blocking Notch with GSI in a metastatic breast cancer model (MDA-231) attenuates bone metastasis by reducing osteolysis in the bone microenvironment. Conversely, overexpression of JAG1 is sufficient to induce bone metastasis in this model (103). Others have demonstrated a role for Notch1 of tumor dormancy in the bone marrow microenvironment, instigating metastases, through a Notch1/STAT3/LIFR signaling axis (134). Furthermore, circulating tumor cells “primed” for breast cancer brain metastases have a specific gene signature (HER2^+^/EGFR^+^/HPSE^+^/Notch1^+^) (135, 136). These CTCs could either be derived from the primary tumor or from metastatic lesions. Importantly, these CTCs were EPCAM^−^. This would make them undetectable by the only FDA approved clinical test for CTCs, which is based on an EPCAM^+^ profile (136).

*Notch2*. Notch2 can act as a transcriptional and functional regulator of Notch1 and Notch3 (137) and has been shown to be involved in specific mammary epithelial lineages affecting luminal cellular hierarchy (138). Mutations in Notch2 show increased incidence in breast cancer, and in addition to the TCGA database new mutations have been found (139). Notch2 is positively correlated with HER2 (140), low-grade tumors and improved outcome (141), and increased apoptosis (142). In the basal subtype, JAG1 and DLL4-induced Notch2 activation under the influence of FYN/STAT5 maintained the mesenchymal-phenotype. Notch2 siRNA decreased the EMT markers VIM, SNAI1, SNAI2 (SLUG), TWIST, and ZEB1 (143). Notch2/3 inhibition (Tarextumab) decreased CSC numbers in the UM-PE13 breast cancer cells (144). Furthermore, mutations in Notch2 could facilitate development of liver metastasis (145). However, other studies showed that, Notch2 mutations do not unequivocally associate with better prognosis and therapy efficacy (144, 146).

*Notch3*. Expression of oncogenic Notch3 in mice leads to mammary cancer (111), and is involved in: hormone-receptor positive breast cancer (120), the proliferation of HER2^−^ breast cancer (147) and HER2^+^ DCIS (148), and TNBC (149). Notch3 is involved in HER2^+^ DCIS through transcriptional upregulation of the Notch pathway by HER2–whereby Notch3 upregulates the formation of luminal cells and increases proliferation through Cyclin-D1, c-MYC, and AKT (148). Furthermore, Notch3 signaling has been proposed to be an important regulator of the process whereby bipotent progenitors commit to the luminal lineage (93). Additionally, evidence from nonsense and missense mutations in multiple cancers, including breast cancer, showed tumor suppressor capabilities of Notch3 through controlling of the cellular senescence pathway (150). Interestingly, no significant change in Notch1, Notch2, or Notch4 expression was observed in these studies.

In TNBC, ectopic NICD3 (over)expression facilitated the inhibition of EMT through upregulation of the HIPPO pathway and E-cadherin in a RBP-jk dependent manner, whereby knockdown of Notch3 abrogated this effect (151). Furthermore, a correlation was shown between Notch3 and p21, a well-known senescence-involved protein. A significant decrease in Notch3 was observed in primary breast cancer, compared with normal tissue, suggesting a protective mechanism against Notch3-initiated cellular senescence. Re-introduction of Notch3 resulted in growth inhibition and activation of cellular senescence, suggesting that loss of Notch3 expression facilitates senescence induction and could play a critical role in tumor progression. Notch3 silencing has recently been shown to sensitize TNBC cells to the EGFR inhibitor gefitinib by promoting EGFR tyrosine dephosphorylation and internalization (152). Notch3/4 were shown to have increased expression in low-burden metastatic cells relative to the primary tumor (153).

*Notch4*. The oncogenic function of Notch4 was first demonstrated by retroviral insertion in MMTV-induced mammary tumors (110). Additionally, Notch4 is highly expressed, and gain of function mutations have been identified, in mouse mammary cancer models [reviewed in (154)]. Expression of activated Notch4 in mammary epithelial cells lead to transformation (155) and rapid development of poorly differentiated adenocarcinoma in transgenic mice (110, 156). Additionally, truncated human Notch4/Int3 (activated Notch4) instigated mammary tumors (112), through transcription of RBP-jk (157) and ANK repeats (158). Interestingly, transgenic expression of Notch4 NICD caused mammary tumors in the absence of RBP-jk in mice harboring conditional knockout of RBP-jk (157). This suggests that non-canonical pathways may participate in the oncogenic activity of Notch4.

*PEST domain*. The PEST domain is a degradation domain that regulates the stability of all NICDs through ubiquitination and proteasomal degradation (39). Nonsense mutations are common in T-ALL (159, 160) and have been observed in Notch1/2/3 receptors in TNBC (39). Furthermore, Notch pathway and target genes, including Notch1/3, HES1, HEY2, HES4, MYC, Cyclin-D1, and NRARP, were highly overexpressed in TNBC (39, 125). Notch mutation-activated dependency was shown using GSI, as wild type tumors showed little to no response (39).

##### Notch pathway-associated proteins

*Fringe*. Fringe is an important regulator of the Notch receptor-ligand interaction (161) through modification (glycosylation) of EGF repeats in the extracellular domains of Notch receptors (162). Fringe enzymes add N-acetyl glucosamine to fructose residues in the extracellular domains of Notch receptors. More glycosylated receptors retain high affinity for Delta ligands but have reduced affinity for Serrate/JAG ligands. Hence, loss of Fringe glycosylation enhances Notch affinity for Serrate/JAG ligands. There are three Fringe genes in mammals: Lunatic Fringe (LF), Manic Fringe (MF), and Radical Fringe (RF). In breast stem or progenitor cells, and especially the terminal end bud cap cells termed “leader cells” (163), LF is highly expressed (93). Conversely, the majority of basal tumors and a subset of claudin-low tumors show reduced LF expression (164). In MMTV-driven tumors, absence of LF exclusively caused triple negative tumors. Furthermore, deletion of LF was enough to cause Notch-driven (Notch1-4) basal-like tumors via enhanced stem/progenitor cell proliferation (163). These tumors resembled “claudin-low” (mesenchymal) subtype of TNBC. p53 loss of function in these tumors resulted in a clear EMT profile (Vimentin, TWIST, E-cadherin) (165). Cells showed increased levels of Vimentin and E-cadherin and decreased expression of cytokeratin 8/14–this coincided with decreased differentiation, increased levels of proliferation, and stem cells. Co-deletion of LF and p53 resulted in upregulated NICD3 and HES5, and downregulation of HES1. These data connect expression of LF, Notch (signaling), and p53 to impaired luminal differentiation.

In contrast to LF, MF is highly expressed in the claudin-low subtype of breast cancer and is associated with Notch4 (166). Deletion of MF shifted the tumor resemblance to a less claudin-low like, more luminal subtype—through increased levels of the luminal marker CK8 and basal marker CK14, and decreased levels of stem cell marker ALDH1. Furthermore, MF was shown to be able to regulate cancer stem cells and their migration in a spheroid model by increasing NICD1 expression and PIK3CG (encoding the g catalytic subunit of PIK3-γ) (166). These data show that Fringe is involved in a Notch-dependent manner in breast cancer with different roles observed for different Fringes (no data has been reported yet on RF)—causing a Fringe-dependent subtype switch (basal-luminal).

*NUMB*. NUMB is a cell fate determinant and endocytic protein that acts as a negative regulator of the Notch signaling pathway (54, 167, 168). NUMB is frequently down-regulated in breast cancer and suppresses the growth of breast cancer cells *in vitro* (169, 170) often involving the attenuation of the p53 tumor suppressor pathway (168). NUMB can drive Notch toward endocytic degradation. Additionally, NUMB inhibits ubiquitin ligase MDM2, which targets p53 for degradation. Hence Loss of NUMB results in a high-Notch, low p53 phenotype. Mechanistically, NUMB forms a ternary complex with MDM2 and TP53 and inhibits the activity of MDM2 (168, 171). In a cohort of breast cancer patients receiving adjuvant chemotherapy, NUMB, and indirectly Notch activation, were inversely correlated with clinical and pathological parameters indicative aggressive disease progression (168). In NUMB-deficient cells, p53 is ubiquitinated and degraded, resulting in chemoresistance and high Notch activity. MDM2 also ubiquitinates NUMB, which results in nuclear translocation and degradation (172). Thus, NUMB connects the MDM2/p53 pathway, the most frequent mutated pathway in human cancers, with Notch signaling.

*MAST*. In many breast cancers, gene translocations and fusions have been described. Recurrent gene arrangements involve MAST and Notch family members (Notch1/2), both showing phenotypic effects in breast cancer (e.g., greater proliferation). Notch fusions were found, almost exclusively, in ER^−^ breast carcinomas. All the fusion transcripts retained the exons that encode for the NICD. Furthermore, higher Notch responsive transcriptional activity was seen in breast cancer cell lines carrying MAST-Notch fusions, and showed dependence on Notch signaling for proliferation and survival (173). The discovery of these Notch fusions warrants further investigation and may identify a biomarker for Notch based therapeutics.

*Nicastrin*. Nicastrin is an essential component of the γ-secretase complex; it encodes an integral membrane protein which associates with the catalytic subunit of γ-secretase, Presenilin (174). Nicastrin is crucial for maturation of Presenilin and cells that lack Presenilin are γ-secretase and Notch-deficient (175, 176). In breast cancer, high Nicastrin is mainly observed in the ER+ subtypes. Nicastrin expression correlates with age and tumor grade–and predicts worse tumor survival (177). Additionally, a set of 22 genes (located on chromosome 1) has been co-identified with Nicastrin amplification and breast cancer (178), however, these genes showed no clear Notch signature. Furthermore, Nicastrin seems to play a role in EMT (177, 179). Targeting of Nicastrin affects breast cancer stem cells and inhibits tumor formation *in vivo* (179). Inhibiting Nicastrin in TNBC, using monoclonal antibodies, showed anti-tumor activity (180). Thus, aiming at Nicastrin provides another opportunity to target the involvement of Notch in breast cancer.

These data suggest that (deregulated) Notch receptor/ligand signaling influences cell renewal in the mammary gland and reaches far beyond mammary development, as it possesses the ability to influence the pre-malignant lesions, primary tumors, the metastatic potential of tumors, and therapy resistance.

#### Notch signaling in the tumor microenvironment

The breast microenvironment consists of a number of cell types including fibroblasts, adipocytes, endothelial and immune cells as well as extracellular matrix.

Cancer associated fibroblasts (CAFs) have been shown to induce Notch activation in breast cancer cell lines through secretion of IL-6 (181). There is also evidence supporting a role for fibroblast-derived microvesicles in endocrine resistance. Cancer-Associated-Fibroblast (CAF)-derived microvesicles, containing oncomiR-221 promoted de novo endocrine resistance—as overexpression of oncomiR-221/222 in luminal breast cancer cells reduces ER expression (182) Furthermore CAFs can promote the cancer stem cell phenotype by secreting CCL2, inducing Notch1 (183). Stromal cells including fibroblasts have also been shown to promote therapy resistance in breast cancer cells through expression of Jagged1 and exosomal transfer leading to Notch3 and STAT1 signaling in cancer cells (184). GPER signaling from both CAFs and cancer cells has been shown to upregulate Notch signaling. 17β-estradiol and GPER ligand G-1 induces γ-secretase-dependent activation of Notch1. Furthermore, the 17β-estradiol and GPER induced migration of breast cancer cells and CAFs is attenuated with GSI treatment (185).

17β-estradiol also promoted increased Jagged1 as well as Notch1 expression in MCF7 cells and was similarly found in endothelial cells. The endothelial cells formed cord-like structures in matrigel in contrast to cells expressing a dominant negative form of Notch1. 17β-estradiol treatment was also able to increase tumor microvessels *in vivo*, which correlated with Notch1 expression (186). Clinical data has shown higher Notch1 activation in tumor endothelial cells compared to non-malignant tissue. A correlation between the rate of NICD1-positve vs negative tumor endothelial cells was higher in patients with positive sentinel lymph nodes (117) Co-culture *in vitro* and *in vivo* has demonstrated upregulation of notch ligands in endothelial cells after contact with breast cancer cells. Proliferation and survival was significantly reduced along with a reduction in the stem-cell population when co-cultures were treated with GSI. Knockdown of Jagged1 in endothelial cells reduced the survival ability of breast cancer cells under starvation conditions. Knockdown also reduced tumor cell proliferation but did not reduce survival of knockdown epithelial cells (187). Wnt signaling is known to be up-regulated in breast cancer. Aberrant wnt signaling has been shown to give a tumorigenic phenotype to primary epithelial cells. This conversion is in part caused by up-regulation of the Notch ligands Dll1, Dll3 & Dll4 which are required for the tumorigenic phenotype (188).

Mammospheres enriched with stem/progenitor cells from node invasive breast carcinoma tissue expressed more IL-6 than matched non-neoplastic mammary glands. Il-6 was only detected in basal-like breast carcinoma tissue which contained stem cell features. Il-6 upregulated Jagged1 and lead to growth and a hypoxia-resistant/invasive phenotype through Notch3 dependent expression of CAIX (189).

Adipocytes within the tumor microenvironment secrete leptin and IL-6. Leptin and IL-6 signaling in breast cancer cells adjacent to adipocytes upregulate multiple pathways including Notch promoting a stem-like phenotype as well as epithelial-mesenchymal transition (190). Leptin is able to induce Notch 1,3 & 4 however Notch3 appears to be cell dependent. The leptin-Notch signaling axis is involved in proliferation and migration and leads to higher incidence and aggressiveness in obese patients. Leptin inhibitors were able to reduce Notch receptor, ligand and target expression (191).

Dll4 and Jag1 have opposite effects on regulating angiogenesis. Jag1 induces maturation of blood vessels, while Dll4/Notch regulates sprouting angiogenesis (192). Thus targeting Dll4 or Jag1 will have different effects. Targeting Dll4 using antibodies promotes non-productive angiogenesis (193). GSI treatment however targets both and leads to a decrease in angiogenesis (194). These differences in targeting may explain the contrasting in angiogenesis seen in pre-clinical models treated with GSI or Dll4 antibodies. In a phase I clinical trial, enoticumab, a Dll4 monoclonal antibody targeting the tumor vasculature, showed stable disease as best response in 2 of the 6 breast cancer patients enrolled. The antibody also gave a number of side effects, seen with previous Notch targeting therapeutics, as well as ventricular dysfunction and pulmonary hypertension (195).

##### Notch and the immune response

The role of Notch signaling in the immune response to tumors is complex and is dependent on the tumor type and microenvironment factors. Notch signaling is a key regulator of hematopoietic development and controls self-renewal, lineage commitment and terminal differentiation of the innate and adaptive immune system including B cells, T cells, myeloid cells, dendritic cells and natural killer cells (196, 197) Notch signaling, both canonical and non-canonical, also plays a role in tumor induced immuno-suppression.

It has been established that most stages of the tumor development from initiation to malignant conversion, invasion, metastasis, therapy resistance and relapse involve the inflammatory response (198). The interaction between tumor cells and immune cells in the tumor microenvironment controls the overall immune surveillance and response to therapies and patient outcome. The role of Notch signaling in the immune response to tumors is complex and is dependent on the tumor type and microenvironment factors Notch as well as regulating many aspects of the immune system regulates many components of the tumor microenvironment (199, 200).

There is a strong causal relationship between endocrine resistance and Jagged NOTCH signaling in breast cancer which promotes macrophage differentiation toward tumor-associated macrophages (TAMs), the most common immune cell found in the breast tumor microenvironment (200). TAMs can be pro or anti-inflammatory depending on micro environmental factors, which in most breast cancers develop the anti-inflammatory phenotype (200, 201). The anti-inflammatory phenotype in breast cancer plays a role in suppressing immune surveillance as well as promoting proliferation, angiogenesis and tissue remodeling (198). In a model of basal-like breast cancer, tumor cells secrete the CCL2 & IL-1β cytokines in a Notch dependent manner, which work to recruit monocytes (202). Within the tumor microenvironment monocytes differentiate into TAMs with a pro tumor phenotype supporting tumor growth and metastasis (203). TAMs also interact with cancer cells via TGFβ, promoting Jagged 1 expression, causing a feedback loop that amplifies cytokine/chemokine secretion.

Myeloid-derived suppressor cells (MDSCs) promote tumor progression through a variety of mechanisms including immune suppression and enhancing angiogenesis and metastasis. MDSCs have been shown to have lower Notch activity in conditioned media from breast cancer cell lines through an inhibitory phosphorylation of NICD by casein kinase 2, disrupting NICD/ CSL interaction (204). MDSCs in breast cancer have also been shown to induce Notch signaling in cancer cells and promote CSC capacity through IL6/STAT3 & Nitric Oxide/Notch cross talk signaling (205, 206). Cancer cells also increase Jagged-1 & Jagged-2 expression in MDSCs leading to a positive feedback loop between cancer cells, immune cells and CSCs.

Notch has been shown to be important in the regulation of Tregs, a subtype of T cells, which is important in peripheral self-tolerance and plays a role in tumor immunosuppression (207). Tregs promote evasion of immune surveillance and are linked to tumor invasiveness and poor prognosis. Notch-1-TGF-β signaling directly induces peripheral Tregs through upregulation of Foxp3 (208). Both Jagged-1 and Jagged-2 increase the generation of Tregs (209) and are highly expressed in TNBC, CSCs and treatment resistant populations (95, 132).

On the other hand CD8^+^ cytotoxic T cells, which have been shown to have anti-tumor function, require Notch to become activated (210), and Notch2 has been shown to be required for the anti-tumor effect of cytotoxic T lymphocytes (211). Furthermore, selective activation of the Notch pathway in hematopoietic environments enhances T-cell activation and infiltration, inhibiting tumor growth in mouse models.

Research into targeting the immune response and the tumor microenvironment is ongoing and detailed reviews strategies and treatments can be found here (212, 213). GSI treatment has been shown to reduce the numbers of TAMs, MDSCs and TRegs, however it can't be excluded that this was in part due to inhibiting tumor growth (214). More research is needed to fully elucidate the complex interplay between Notch, tumor microenvironment and the immune system in breast cancer and to develop strategies that enhance the anti-tumorigenic effect but do not suppress the anti-tumor immune response.

## Notch in breast cancer therapies

### Radiotherapy

For breast cancer, radiotherapy is mainly implemented in the adjuvant setting and involves the targeting of remaining tumor cells, with the aim to prevent recurrence of residual disease. Gene signatures (IGKC, RGS1, ADH1B, DNALI1) in primary breast cancers predict low and high risk groups for local regional recurrence after Radiotherapy (215, 216). Generally, cancer stem cells are often radiation resistant (217, 218). Radiotherapy resistance could be intrinsic or acquired through changes in gene expression profiles and radiotherapy-resistant CSCs have been observed in breast cancer (219, 220). More specifically, BCSCs (CD44^+^/CD24^−/low^) were shown to be resistant to radiation (compared to non- CD44^+^/CD24^−/low^ mono-layer cultures), and contributed to tumor recurrence after fractionated radiation. In a clinically more relevant culture system (mammospheres) higher radiation resistance was observed correlating with lower levels of ROS compared to monolayer cultures. Consistently, mammosphere cultures showed higher radiation resistance than irradiated single cell suspensions. Thus, during fractionated radiation, repopulation derives from the more resistant subpopulation of CSCs. Increased levels of Notch1/JAG1 signaling could stimulate the more resistant phenotype of CD44^+^/CD24^−/low^ CSCs (219). Lagadec et al. showed that radiotherapy-exposed cancer cells have increased mammosphere formation, increased tumorigenicity, and (re)expressed stemness-related genes (transcription factors Oct4, Y-box 2, Nanog, and Klf4). Interestingly, both NICD1 and JAG1 expression were upregulated only in response to fractionated radiation (5 × 3Gy) and not after a single dose (10Gy) (221). Additionally, other research showed that a singular dose of 3Gy did upregulate NICD1 and JAG1 (222). Thus, induction of Notch pathway genes is radiation (multi)dose-dependent (222). Furthermore, targeting of Notch using siRNA (221) or GSI (222) decreased the induced BCSCs population after irradiation of non-tumorigenic cells. These data indicate that Notch is involved in the induction of radiation-induced CSCs from partially differentiated tumor cells. Recently it has been shown that, that Notch1 directly regulates the DNA damage response, through physical interaction and suppression of phosphorylation of ATM kinase (223). A plausible hypothesis is that after repeated irradiation, Notch1 could suppress apoptosis-inducing signals from the activated DNA damage response.

### Chemotherapy

Chemotherapy is an important component of standard cancer treatment and includes anthracyclines, cyclophosphamide, and taxanes. Resistance to chemotherapy is the main cause of treatment failure in 90% of the patients with metastatic cancers (224). Importantly, chemo-resistance accompanies endocrine resistance, so that ER-positive recurrent tumors that are resistant to endocrine therapy are also almost invariably chemo-resistant. One of the main underlying causes for treatment failure is intra-tumor heterogeneity, a process affected by the presence of CSCs (168, 225).

#### Anthracycline/cyclophosphamide

A role of Notch in doxorubicin sensitivity and resistance has been reported by Zang et al. (226). They showed that Notch1 inhibition (RNAi) and doxorubicin treatment led to a 50 and 70% growth inhibition, and increased apoptosis, compared to chemotherapy alone–in the MCF7 and MDA-MB-231 cell lines respectively. Li et al. showed that the efficacy of doxorubicin could be increased when used in combination with a GSI (227). Additionally, chemotherapy increases the percentage of treatment resistant CD44^+^/CD24^low^ breast cancer cells in patients. In tumor xenografts combination treatment with GSI and doxorubicin led to better tumor control–by reducing CD44^+^/CD24^low^ population (168).

Interestingly, ALDH expression has been shown to inactivate chemotherapeutics such as doxorubicin and cyclophosphamide (228–230). In addition, Suman et al. (231) showed that Notch inhibition was effective in both ALDH^−^ and ALDH^+^ cells, though ALDH^−^ cells were more sensitive. Additionally, they showed that Notch1 downregulation (using Psoralidin) and silencing resulted in inhibition of cell viability and proliferation, and a downregulation of EMT factors SLUG and TWIST.

In ER+ cell lines (MCF7 and T47D) Notch target genes HES1 and HEY1 were induced by doxorubicin, and could be inhibited using a GSI–suggesting a Notch signaling dependent effect. Furthermore, expression of Notch was associated with expression of multi drug resistance protein 1 (MRP1), a potential predictor of chemotherapy response and clinical outcome, in a dose-dependent manner (232). Importantly, in patients treated with neoadjuvant chemotherapy (anthracyclines ± taxanes), pre-treatment NICD1 levels were very low or absent, while post-therapy NICD1 was significantly upregulated (232).

In a doxorubicin resistant engineered-cell line, MCF7-AMD, Notch3 was shown to be downregulated in chemo-resistant cells, and EMT was activated. Furthermore, in ER^+^ patients, low Notch3 predicted distant relapse-free survival, with Fos-related antigen 1 (Fra1) being negatively regulated by Notch3 (233).

#### Taxanes

The two most common used taxanes for breast cancer treatment are docetaxel and paclitaxel (234, 235). Qiu et al. showed that docetaxel treatment resulted in increased primary mammosphere formation. Notch1 inhibition increased chemotherapy efficacy in TNBC BCSCs (CD44^+^/CD24^−/low^ population) *in vitro* and in a patient-derived xenograft breast cancer model (236). In line with this, Zhang et al. reported similar findings, using a GSI in multiple xenograft models (237). “Tumor debulking” by docetaxel resulted in an increased BCSC population, quantified using ALDH^+^/CD133^+^/CD44^+^. Interestingly, the CD44^+^/CD24^−/low^ population was not altered, however, this might be due to differential targeting methods (Qiu et al. (236): mAb vs. Zhang et al. (237): GSI). Docetaxel-treated tumors showed increased NICD1. Combination of GSI with docetaxel showed significant improved effect compared to docetaxel alone. Mechanistically, treatment with docetaxel caused an increase in survivin (inhibitor of apoptosis) and drug transporters, which could be inhibited by GSI. Furthermore, decreased expression of NUMB was observed in docetaxel treated tumors but not after dual treatment with GSI. Docetaxel treatment increased EMT markers SNAIL, SLUG and N-cadherin, which could be blocked by Notch inhibition. These findings indicate that Notch1 is involved in the resistance mechanisms of docetaxel treated tumors and that dual treatment could block enrichment of the BCSC population and increase therapy efficacy.

Schott et al. showed a residual BCSC subpopulation to be insensitive to docetaxel alone (238). However, in tumor-derived xenografts treatment with GSI (MK-0752) reduced the BCSC population; this resulted in reduced mammosphere formation and decreased NICD and HES1 expression. A concurrent clinical study, including 30 patients with recurrent disease after anthracycline treatment, showed that repeated cycles of GSI resulted in partial response in 11 patients and evidence for a reduction in CD44^+^/CD24^−/low^ and ALDH^+^ cells. Repeated biopsies showed an initial increase in BCSC populations until after the 1st treatment cycle, after which it declined—this is consistent with the ability of GSIs to decrease BCSCs. However, additional treatment cycles where needed to additionally reduce BCSCs and tumor burden. An additive effect of Notch inhibitors and docetaxel has been recently observed in a phase 1b trial in TNBC, whereby docetaxel and GSI (PF-03084014) showed 4 partial responses and 9 had stable disease out of 25 patients, with a manageable safety profile (by dose reduction) (239). All in all, the combination of docetaxel and Notch1 targeting showed synergy, with a manageable toxicity profile (238, 239).

In TNBC cells treated with the microtubule stabilizing agent paclitaxel, surviving breast cancers cells expressed Notch1, Sox2, Oct3/4, c-Myc, c-SRC, c-MET, Nanog, and E-cadherin, and were highly tumorigenic. Surviving cells also became resistant to the BCR-Abl/Src family kinase inhibitor dasatinib (240). In parental MDA-231 cells, dasatinib reduced NICD1 and Cyclin-D1 levels, but in paclitaxel resistant clones NICD1 levels were not affected. Dasatinib resistant MDA-231 clones were not cross-resistant to doxorubicin or docetaxel. Targeting Notch1 signaling in TNBC (using GSI) was additive to paclitaxel treatment, as Notch wildtype tumors showed no additive effect (125). These results support a protective mechanism whereby Notch1 is upregulated to protect the survival of paclitaxel-treated TNBC cells. In the TNBC UM-PE13 xenograft, blockage of DLL-4, decreasing Notch1 signaling, resulted in delayed tumor regrowth after paclitaxel treatment, with additionally decreasing the CSC frequency (241). Paclitaxel is capable of preventing breast cancer bone metastases. However, resistance emerges over time through induction of osteoblast JAG1 expression. Hence, metastatic seeding could be prevented using a JAG1 antibody (15D11). Synergistic effects (100x, compared to IgG) were observed when used in combination with paclitaxel (242).

All together, these data indicate that Notch inhibition may sensitize breast cancer to chemotherapeutics and that this involves a treatment-resistant BCSC population characterized by CD44^+^/CD24^−/low^ cells. Further, chemotherapy resistant cell lines may be resensitized after treatment with Notch inhibitors.

### Endocrine therapy

In ER^+^ breast cancers, estrogen receptor signaling plays a pivotal role in tumor development and progression (243). Treatments that target the ER include blocking of receptor with an antagonist (e.g., selective estrogen receptor modulators such as tamoxifen or selective estrogen receptor disruptors such as fulvestrant), or depriving the tumor of estrogen (aromatase inhibitors). This mainly targets the tumor bulk, however, important implications have been made for hormone receptor-positive stem cells (244, 245). Despite similar expression of hormone receptors, some tumors are more sensitive to endocrine therapy than others, resulting in inter- and intra-patient differences. Additionally, differences in clinical outcome are observed based on breast cancer subtype (Table 1). Notably, expression of ER/PR is not universal in both tumor and metastases (246), and this does affect tumor prognosis (247). Receptor conversion and intra-tumor heterogeneity of ER expression in primary and metastatic tumors are therefore still a barrier to effective endocrine therapy. Point mutations in the ESR1 gene, encoding ERα, have been shown to arise during endocrine therapy and lead to endocrine resistance (248, 249).

#### Notch, estrogen receptor interactions, and therapy sensitivity/resistance

It has been suggested that in endocrine-resistant tumors, the ER is not the main survival pathway of breast cancer cells. Additionally, ER-targeting treatment resistance mechanisms are already in place (250, 251), and these resistance mechanisms show potential activating crosstalk with Notch (57). Endocrine resistant breast cancers show increased BCSCs numbers (9, 252) with Notch3/4 expression (94, 252, 253). Interestingly, in BCSCs paracrine EGFR and Notch signaling (under the influence of estrogen), is capable of activating estrogen signaling in ER^−^ BCSCs (253).

Estradiol inhibits the activation of Notch1/4, causing membrane accumulation of uncleaved receptors (254), and upon estrogen deprivation or anti-estrogen drugs increased Notch signaling was observed (254). Luminal breast cancers with Notch1 remain hormone responsive (9). Hence, decreasing Notch signaling using GSI in cell lines and xenografts resulted in G_2_ growth arrest (254). Additionally, estrogen deprivation of luminal ER^+^ cells (MCF-7) inhibits tumor growth. Conversely, in the engineered HER2^+^ MCF-7 cell line, tamoxifen stimulated growth, even in combination with estrogen deprivation. This was accompanied by molecular crosstalk between ER and HER2 (255). Furthermore, involvement of the Akt and MAPK pathways were observed, with possible roles for Notch in this resistance (59, 256, 257). These experiments indicate that HER2 expression plays an important role in endocrine therapy resistance mechanism; however luminal cells are still dependent on estrogen receptor activation.

Interestingly, when grown orthotopically, original ER^+^/PR^+^/CK5^−^ tumors showed an increased population of ER^−^/PR^−^/CK5^+^ “luminobasal cells,” this population further increased when estrogen was withdrawn, revealing receptor conversion when exposed to a new environmental niche (9). However, others have stated that this ER^−^/PR^−^/CK5^+^ population doesn't increase over time, is under the influence of progesterone signaling, and is capable of surviving extensive ER-targeting (258). Many Notch1 pathway genes were included in this new so-called luminobasal gene signature—involving TWIST1 and SLUG upregulation. These luminobasal cells resemble a more TNBC basal-like phenotype (CK5^+^) while retaining their luminal origin, expand (at higher rates) within luminal tumors when deprived of estrogen signaling due to their independence of the estrogen receptor, and showed sensitivity to Notch1 silencing. These data suggest an important link between ovarian endocrine sensitivity (both progesterone and estrogen) and Notch1, and support a luminal origin of basal-like cells (9, 258).

Elevated Notch1/3 signaling upregulates IL6 and activates the JAK/STAT pathway, however, dependent on p53/IKKa/IKKb status, and through a non-canonical mechanism. Furthermore, Notch signaling upregulation resulted in different Notch target genes in different molecular subtypes of breast cancer (basal vs. luminal B) (259). This growth promoting effect can also be instigated by fibroblasts secreting IL6, in relation with Notch3 and JAG1 (181). Pioneering research by Sansone et al. showed that Notch3-IL6 signaling is under indirect control of hypoxia and that it promotes self-renewal and survival in mammary gland stem cells (260, 261). CD133^high^ cells express low levels of ER, but high levels of Notch3 (252), are endocrine resistant and promote metastases. This process is regulated through IL6-Notch3 signaling (261). IL6 expression could be induced either by Tamoxifen or HER2. CD133^high^ expressing cells could be resensitized to endocrine therapy through IL6R blockade, which reduced Notch3, STAT3, and CD133. Knockdown of STAT3 resulted in reduced Notch3 mRNA levels and re-expression of ERα, without changes in CD133 expression. Notch3 thus, indirectly, plays an important role in endocrine resistance observed in metastatic breast cancer by influencing stem cell behavior (260, 261).

As described earlier fibroblast-derived microvesicles containing oncomiR-221 promoted *de novo* endocrine resistance—as overexpression of oncomiR-221/222 in luminal breast cancer cells reduces ER expression (182). These microvesicles were capable of blocking endocrine therapy Notch3 down regulation and causing an estrogen-independent phenotype in breast cancer cells (96, 262). This was also observed in endocrine resistant luminal breast cancers whereby blockage of Notch3 abrogated the growth of these ER-resistant cells (262).

Moreover, Notch4 is a crucial mediator of endocrine therapy resistance in models of luminal breast cancers (95, 261–263). BCSC induced by endocrine treatment are characterized by upregulation of Notch target genes [and additionally induces an EMT phenotype (263)], and endocrine resistance in BCSC is driven through JAG1/Notch4 signaling (95). This could be inhibited through targeting of Notch4 using GSI RO4929097. Notch4 inhibition reduced HES1 and HEY1 expression, reversed EMT, decreased CSC populations, thereby attenuating proliferation and invasion. Notch4 thus promotes estrogen-independent, endocrine therapy resistant growth of breast cancer cell lines (95, 263) possibly through a Notch4/STAT3/EMT regulated axis (264). Very recent evidence shows that mutations in the ligand binding domain of ERα, which occur in patients and are associated with endocrine therapy resistance, promote a stem-cell-like phenotype through activation of Notch4 (265).

### Targeted therapy (HER2)

HER2, a family member of the ERBB transmembrane receptor tyrosine kinases (ERBB1-4 or also known as EGFR and HER2-4) is a well-known target in HER2-amplified breast cancer therapy for both primary tumors (266, 267) and metastases (268–270). However, it is still unclear whether HER2^+^ cells are truly addicted to oncogenic HER2 signaling as other EGFR members can compensate after HER2 blockade (266). Moreover, a single copy of HER2 (in the absence of genomic amplification) can elicit an expression signature associated with HER2 dependence. Thus, HER2-non-amplified tumors may in some cases benefit from HER2 targeted therapy. Yet, such tumors are currently not being selected for treatment (267, 271). HER2^+^ breast cancer is mainly treated with combinations including taxane-based chemotherapy plus trastuzumab (272), pertuzumab (273), the tyrosine kinase inhibitor lapatinib (274, 275), or combinations thereof (266, 276). Many trials have shown remarkable response rates (277–281), and therefore HER2-targeted therapy is standard of care. However, intrinsic and acquired resistance may still result in relapse and progression of HER2^+^ disease. This resistance can occur on many levels, including activation of the downstream signaling pathways, constitutively activated HER2, and crosstalk of HER2 with other growth factor receptors such as other EGFR-members and IGF (282–285).

HER2 is a direct Notch target gene and bidirectional crosstalk between Notch and HER2 has been extensively reviewed (286). Under trastuzumab treatment, Notch activation occurs and contributes to trastuzumab resistance (284, 287). Trastuzumab-resistant cells (treated with trastuzumab for 6 months) expressed higher levels of Notch pathway genes, and this could be reversed by Notch inhibition (siRNA). GSIs decreased proliferation (288). In HER2+ xenograft experiments, GSI MK0752 alone did not affect tumor volume, while trastuzumab alone caused complete regression of tumors. However, trastuzumab-treated tumors recurred in approximately 50% of the cases. When trastuzumab was combined with GSI MK0752, complete cures were obtained with no observed recurrences. This suggests that the combination trastuzumab/GSI targeted stem-like cells responsible for recurrent disease. Notch inhibition resulted in HER2 down regulation (under the influence of Notch/RBP-jk binding sites in HER2 promotor sequences), followed by decreased mammosphere formation (286, 289).

Furthermore, Notch signaling is upregulated after treatment with lapatinib, a clinically active small molecule EGFR/HER2 inhibitor. Blockade of HER2 signaling in HER2-dependent primary tumor cells led to upregulation of Notch signaling [NICD1, HEY1, and HEY2 (266)]. The feedback signaling between these pathways was confirmed by the ability of HER2 to represses Notch signaling through HES1 and NRARP. In a HER2-inducible mouse model, Notch1 gain-of-function constructs identified Notch dependency in tumors recurring after suppression of HER2 expression in an HER2 inducible mouse model. After HER2 removal, the rate of recurrence was much higher in primary tumors that overexpressed NICD1, and this could be blocked using GSIs (118). The GSI sensitivity of these tumors suggests that other wild-type Notch paralogs (e.g., Notch3) induced by NICD1, may play a role. Moreover, a meta-analysis (17 studies, including 4,463 patients) revealed increased Notch activity in a subset of breast cancers associated with poor clinical outcomes (including basal-like tumors). These data suggest that Notch is positively associated with tumor recurrence in breast cancer patients and implicate that Notch targeting might prevent recurrent disease by targeting the dormant residual tumor cells.

Interestingly, HER2 expression can be heterogeneous both in bulk tumor cells (290) and BCSCs (291), and shows plasticity (291). ER^+^/HER2^−^ and TNBC acquire a HER2^+^ subpopulation following therapy exposure (267, 291). Cultured BCSCs from ER^+^/HER2^−^ patients retained HER2^+/−^ subpopulations and switching between these HER2 states is dependent on environmental stimuli (291). Notch was inversely correlated with HER2 expression and HER2^−^ cells were sensitive to Notch inhibition. HER2^+^ cells showed higher proliferation but were not addicted to HER2 oncogenic signaling. Following these sub-profiles, a proliferative state/niche favored the HER2^+^ phenotype, whereas oxidative stress or chemotherapy selected for, or initiated transition to, HER2^−^ BCSCs. Thus, Notch might mediate a protective mechanism by functioning in the switch between proliferative and survival-prone phenotypes of HER2^+/−^ BCSCs.

Besides a direct link between Notch and HER2, Notch also interacts with downstream or parallel HER2 signaling pathways. Co-suppressing the activation of these pathways upon resistance (267) might bypass these resistance mechanisms. Alternative mechanisms to activate signaling pathways such as PI3K/AKT and/or MAPK can be triggered in response to trastuzumab or through constitutive activation of HER2. These pathways may mediate treatment resistance in selected clones. The communication between Notch and PI3k/AKT has been shown extensively in hematological cancers (59, 292) and to lesser extent in breast cancer (293). Bidirectional MAPK-Notch interactions have been described (256, 257). Additionally, when the HER2 receptor is inhibited, signaling might still occur due to dimerization with IGF1-R (285) and Notch interaction (294), possibly resulting in therapy resistance.

## Discussion, conclusion, and future perspectives

There is overwhelming evidence for a role of the Notch signaling pathway in breast cancer development and progression through upregulation of Notch receptors, ligands, and regulators. Overall, high Notch pathway activity is associated with more aggressive disease and poor outcomes. Only a limited number of breast cancers harbor Notch gain of function mutations, but in many breast cancers Notch is expressed, active, and crosstalks with other oncogenic pathways. Further, many studies support an important role for Notch in the response to radiation, chemotherapy, hormonal therapy, and targeted therapies. Importantly, there is compelling evidence that treatment-resistant breast cancer and other malignancies can be resensitized by Notch inhibition (77, 78, 295–297). Taken together, this provides a strong rationale for studies combining Notch inhibitors with current breast cancer treatment modalities.

However, an important and complicating feature of Notch signaling is its receptor-ligand specific and context dependent signaling in different cancer subtypes. Furthermore, the optimal timing to initiate treatment to achieve therapeutic efficacy must be carefully considered. In treatment-naïve tumors, Notch activation might not become clinically evident until treatment initiation, as a resistance mechanism triggered by treatment, or after occurrence of metastases with different mutational profiles compared to primary tumors (4). Selecting patients most likely to benefit from Notch inhibition will require molecular profiling and screening to show possible co-targeting options (298). The identification of predictive biomarkers is of paramount importance.

In this review, we have highlighted several opportunities for Notch targeting in the context of first line breast cancer treatment and resistance. Additionally, we have discussed its extensive communication with many other pathways (59, 256, 257, 292, 293), its role in recurrent disease and involvement in the metastatic process (103, 134, 136, 145), and its association with clinically relevant hallmarks in breast cancer (69).

Research in the past decade has focused on preventing or treating tumor recurrence by targeting CSCs. Multiple different stem-like cell populations have been proposed within tumors, based on the expression of CD44^high^/CD24^−^, ALDH^+^, CD133, CD29^high^/CD61^+^, CD49f^+^, and CD90 (299–302). These cells showed increased levels of therapy resistance and distinctive gene expression patterns, irrespective of their potential origin (e.g., from transformation of mammary stem cells or from de-differentiation of non-stem-like tumor cells)—as stem cell plasticity occurs within tumors (32, 303). Notch signaling plays an important role in mammary stem cells as well as breast cancer stem cells (BCSCs) (84, 92, 304)—well documented for triple-negative breast cancer (94, 109, 153, 244, 253, 304–307). Furthermore, Notch4 has been shown to maintain the BCSC population (94, 307). Notch-PTEN signaling is important in the expansion of these stem-like cells (98, 308). PTEN/PIK3CA mutations are often observed in breast cancer and loss of PTEN decreases radiation sensitivity (309). In the future, combining radiotherapy and small molecule targeting in BCSC may improve the efficacy of radiation therapy and forestall radiation resistance. However, the timing and sequencing of treatments should be carefully optimized in order to achieve maximum efficacy. Radiotherapy dose scheduling might be easily adapted from the current schedule standards (310–312).

The effects of chemo-, radio- and targeted therapy on Notch signaling require further investigation. Observations have been made for Notch and tumor vascularization under the influence of both anthracycline and taxane-based chemotherapy (313). Taxane (paclitaxel) therapy resistance coincides with the development of metastatic bone lesions, preventable by targeting JAG1 in osteoblasts (242).

BCSCs in ER+ tumors show responsiveness to hormone signaling/targeting despite often lacking ER and PR (244, 245). This may be mediated by paracrine crosstalk with ER^+^/PR^+^ bulk tumor cells. Many endocrine therapy resistance mechanisms have been revealed (314–317). This has guided research toward the development of new therapeutic regimens (318), such as CDK4/6 inhibitors (319)—which have been clinically implemented. Notch inhibition could play a significant role in combinations targeting these resistance mechanisms. For instance, Notch inhibition could reverse ER-targeted-treatment resistance and improve the efficacy of CDK4/6 inhibitors through decreasing Cyclin-D1 (121).

Notch has been shown to crosstalk with the HER2 receptor (289) and development of breast cancer metastases is affected by HER2 (268–270, 320) and progesterone (268). Interestingly, plasticity of HER2 expression has been observed in circulating tumor cells—with a distinctive role for Notch1 (291). Thus, Notch is involved in the heterogeneity and plasticity observed in HER2^−/+^ breast cancer, and the development of distant metastases. Combining CDK4/6 inhibitors (321, 322) and Notch inhibitors, it may be possible to simultaneously attenuate two main drivers in breast cancer, HER2 and Cyclin-D, promoting local control and preventing distant relapse.

A step forward, for individualized patient care, could be the use of patient-representative culture models, such as organoids, to capture information on individual tumor drug sensitivity *ex-vivo* (323). In general, organoids can provide rapid insight into individual treatment combinations and relationships between Notch signaling and breast cancer treatment (resistance), before the start of treatment. These models more closely represent individual tumors, and may enable us to rationally investigate the context-dependence of Notch signaling in each tumor. Breast cancer organoids have recently been developed, but to what extent they will be strong predictors of treatment response and their use as prospective platforms for individualized precision treatment remains to be established (324).

**Figure 3:**
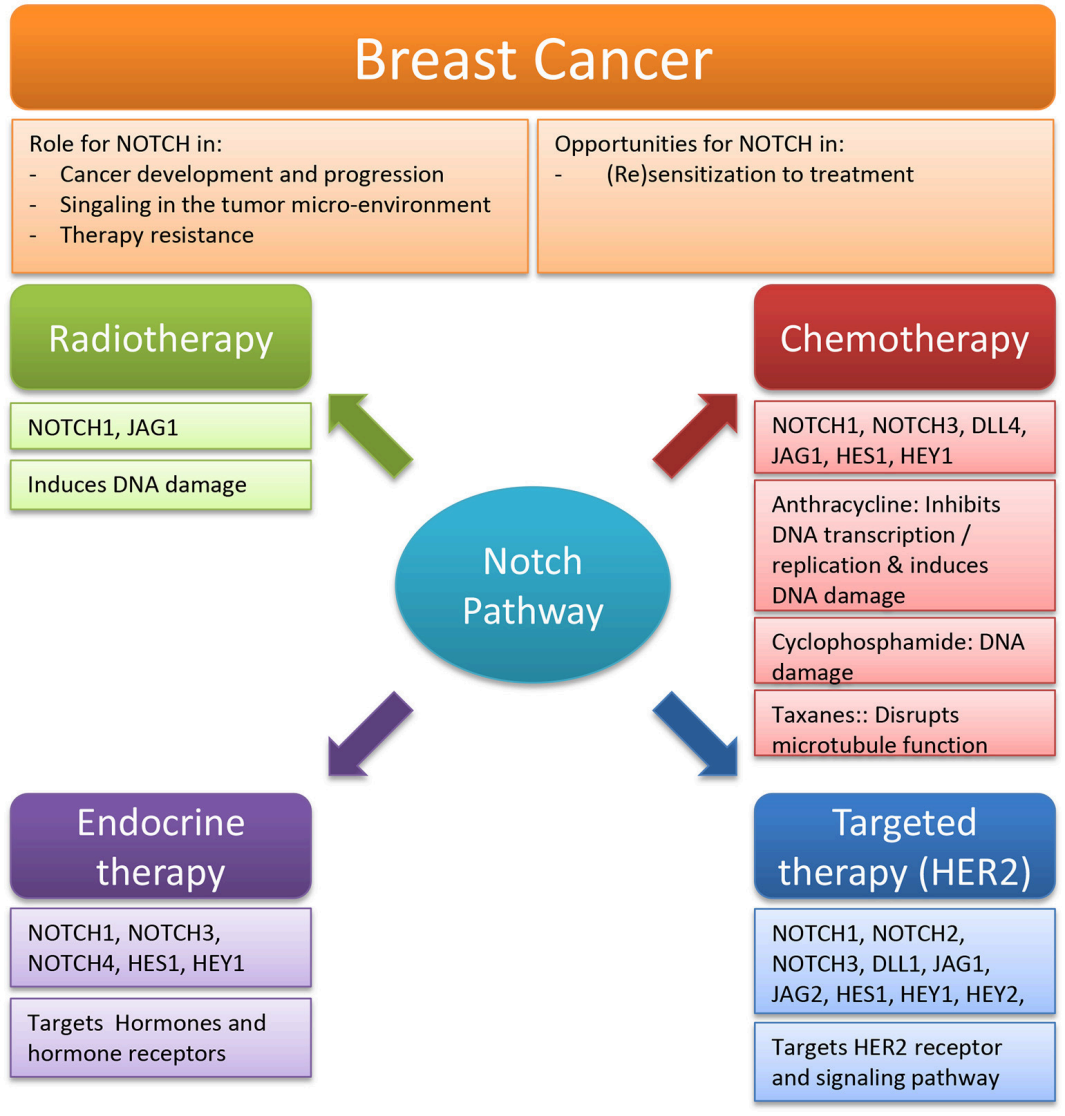
Overview of the role and opportunities for Notch in breast cancer therapy. Summary of the 4 fields of breast cancer therapy [radiotherapy, chemotherapy, endocrine therapy, and targeted therapy (HER2)] in which Notch targeting can play a significant role.

This review summarizes the evidence supporting the hypothesis that targeting Notch could a promising option in re-sensitizing breast cancer to current standard of care treatments (Figure 3). When biomarker quantification and patient stratification allow Notch targeting to live up to its potential, this strategy may be applicable to other cancers as well, targeted with concurrent chemo-radiation or targeted inactivation of other growth promoting pathways. However, clinical evidence in solid tumors showed that therapy timing is highly important to reach maximum effectivity (325). Thus, additional clinical and translational research will be required to determine the exact role of Notch in each disease- and treatment-specific context and fine-tune the use of Notch targeting agents to prevent or treat or acquired resistance. With the benefit of sufficient mechanistic knowledge, we propose that in some cancer patients targeting Notch can be a major part of an effective strategy to address therapy resistance.

## Author contributions

EM, JI, MS, and MV made substantial contributions to conception and design of the review, and analysis and interpretation of articles. All authors have been involved in drafting the manuscript or revising it critically for important intellectual content. All authors have given final approval of the version to be published. EM, JI, MS, and MV have agreed to be accountable for all aspects of the work in ensuring that questions related to the accuracy or integrity of any part of the work are appropriately investigated and resolved.

### Conflict of interest statement

The authors declare that the research was conducted in the absence of any commercial or financial relationships that could be construed as a potential conflict of interest.

## Abbreviations

(B)CSC: Breast Cancer Stem Cell
ANK: Ankyrin Repeats
CAF: Cancer Associated Fibroblast
DCIS: Ductal Carcinoma *in situ*
EGF: Epidermal Growth Factor
EMT: Epithelial-Mesenchymal Transition
ER: Estrogen Receptor
GSI: Y-Secretase Inhibitor
HER2: Human Epidermal Growth Factor Receptor 2
JAG: Jagged
LF: Lunatic Fringe
LNR: Lin12-Notch Repeats
LRR: Loco-Regional Recurrence
MAML: Mastermind-Like
MaSCs: Mammary Stem Cells
MDSC: Myeloid-derived suppressor cell
MET: Mesenchymal-Epithelial Transition
MF: Manic Fringe
MMTV: Mouse Mammary Tumor Virus
MRP1: Multi Drug Resistance Protein 1
NICD: Notch Intracellular Domain
NLS: Nuclear Localization Sequences
NRR: Negative Regulatory Region
pCR: Pathological Complete Remission
PR: Progesteron Receptor
RAM: RBP-Jk Association Module
RF: Radical Fringe
TAM: Tumor associate macrophage
TCGA: The Cancer Genome Atlas
TN(BC): Triple Negative (Breast Cancer).
